# Potential of Circulating MicroRNA Panels to Discriminate Peripheral Arthritis in the Spondyloarthritis Spectrum: A Preliminary and Validation Study

**DOI:** 10.3390/medicina62071314

**Published:** 2026-07-08

**Authors:** Ching-Fu Huang, Jim Jinn-Chyuan Sheu, Yu-Jih Su, Chung-Yuan Hsu

**Affiliations:** 1 Division of Cardiology, Department of Internal Medicine, Kaohsiung Municipal Ta-Tung Hospital, Kaohsiung 833, Taiwan; 2 Institute of Biomedical Sciences, National Sun Yat-sen University, Kaohsiung 804, Taiwan; 3 Division of Rheumatology, Allergy and Immunology, Department of Internal Medicine, Kaohsiung Chang Gung Memorial Hospital, Chang Gung University College of Medicine, Kaohsiung 833, Taiwan; 4 School of Medicine, College of Medicine, National Sun Yat-sen University, Kaohsiung 804, Taiwan; 5 Center for Mitochondrial Research and Medicine, Kaohsiung Chang Gung Memorial Hospital, Kaohsiung 833, Taiwan

**Keywords:** autoantibody, biomarker, microRNA (miRNA), NF-κB pathway, psoriasis without clinical arthritis (PsO), spondyloarthropathy with peripheral arthritis (p-SpA)

## Abstract

*Background and Objectives:* The clinical differentiation of peripheral involvement within the spondyloarthritis (SpA) spectrum remains a significant challenge. Identifying patients at the stage of psoriasis without clinical arthritis (PsO), before the onset of overt arthritis, is crucial for early disease management. MicroRNAs (miRNAs) have emerged as potential epigenetic regulators in inflammatory rheumatic diseases. This study aimed to identify circulating miRNA profiles that serve as discriminative biomarkers between PsO and peripheral SpA (p-SpA). *Materials and Methods:* This exploratory study was conducted in two phases. In the preliminary discovery phase, plasma miRNA expression was analyzed using high-throughput microarrays in patients with p-SpA (modeled by peripheral psoriatic arthritis, *n* = 6), PsO (psoriasis without clinical arthritis, *n* = 3), and osteoarthritis (*n* = 3). In the validation phase, candidate miRNAs were verified using TaqMan-based qPCR in an independent cohort (*n* = 30) of various SpA phenotypes, categorized into those with peripheral arthritis (SpA-A) and those without (SpA-N). *Results:* The preliminary discovery phase identified altered levels of hsa-miR-140-5p, hsa-miR-192-5p, and hsa-miR-146a-5p among the groups; however, due to the small sample size, these initial findings required strict downstream verification. Functional enrichment analysis revealed that these candidate miRNAs primarily targeted the NF-κB signaling pathway (hsa04064) and Toll-like receptor (TLR) signaling pathway (hsa04620). The validation cohort confirmed that these three miRNAs could reliably differentiate SpA-A from SpA-N patients. Furthermore, bioinformatic mapping predicted that downstream targets, including *TRAF6*, *IRAK1*, and *CXCL2*, may be associated with these clinical phenotypes, serving as hypothesis-generating observations for future studies. *Conclusions:* Our findings suggest that specific plasma miRNA profiles are associated with the inflammatory pathways driving peripheral involvement in the SpA spectrum. These miRNAs represent potential biomarkers associated with peripheral arthritis in the SpA spectrum. While they offer new molecular insights into disease pathogenesis, their predictive value for identifying PsO patients at risk of developing overt arthritis requires confirmation in future longitudinal studies.

## 1. Introduction

Psoriasis is a multisystemic disease that involves not only the skin [1,2] but also the joints [3,4,5,6], nails [7], kidneys [8], gastrointestinal system [9,10], cardiovascular system [11], and heart [12], and it may be related to the development of cancer [13]. In this study, we investigated psoriasis patients without clinical joint involvement (the PsO group) to explore the molecular transition to overt arthritis.

A previous large retrospective study using the National Insurance database indicated that psoriasis is associated with dozens of comorbidities compared with normal controls [14,15]. A recent study reported that the prevalence rate of peripheral arthritis in spondyarthropathy (p-SpA), manifesting as psoriatic arthritis, is approximately 17% among patients with psoriasis in dermatology clinics [16]. Previous studies have shown that PsO and p-SpA are difficult to diagnose on the basis of serological findings. Only one report has focused on the use of autoantibodies against extractable nuclear antigens in PsO [17]; however, several have concentrated on comparing the molecular profiles between patients with PsO and those with p-SpA. In fact, it was not until recently that a detailed study of genetic risk factors associated with the risk of p-SpA among psoriasis patients became available [18].

The early detection and diagnosis of p-SpA pose a challenge to rheumatologists [19], even when aided by sonography [20]. The Madrid Sonographic Enthesitis Index (MASEI) has a sensitivity of only 30% for diagnosing p-SpA. An update of the treat-to-target concept by a Canadian dermatologic expert suggested looking beyond the skin [21]. Such treat-to-target concepts require the early diagnosis and treatment of p-SpA [22]. Therefore, we are looking for a pathogenesis-oriented method to differentiate PsO from overt p-SpA patients.

Epigenetic regulation by miRNA networks is key to controlling physiological and pathological conditions of the immune system [23,24]. Each miRNA represents one or more post-translational modifications in gene transcription and mRNA translation. Differential expression of certain miRNA panels may be associated with different clinical characteristics between PsO and p-SpA, potentially serving as serological markers to improve diagnostic tests for the early identification of p-SpA in patients initially without clinical arthritis.

Given the critical role of circulating miRNAs in regulating cellular functions and immune responses, identifying specific miRNA signatures could provide valuable insights into the heterogeneous pathogenesis of the spondyloarthropathy (SpA) spectrum. While clinical assessment remains the cornerstone for identifying peripheral involvement, there is a significant unmet need for molecular biomarkers associated with peripheral arthritis, particularly to differentiate patients with psoriasis without clinical arthritis (PsO) from those with overt p-SpA. Therefore, the present study was designed as a two-phase investigation to explore the discriminative potential of plasma miRNA profiles. In the initial discovery phase, we utilized a high-throughput microarray to screen for differentially expressed miRNAs between PsO and p-SpA patients. This was followed by a validation phase in an independent SpA cohort to verify the identified candidates and their associated inflammatory pathways, specifically the NF-κB and Toll-like receptor (TLR) signaling pathways. By characterizing these molecular signatures, we aim to bridge the gap in our understanding of the transition from cutaneous disease to peripheral joint involvement in the SpA spectrum.

## 2. Materials and Methods

### 2.1. Patients

This prospective study recruited patients from three different groups: patients with p-SpA, PsO, and osteoarthritis (OA) as a control group. Patients with OA were specifically selected to represent a non-systemic, mechanically driven joint disease. This control group allowed us to filter out background miRNA expression associated with general joint degeneration and non-specific damage, thereby isolating the miRNA signatures uniquely driven by the systemic inflammatory pathogenesis of the SpA spectrum. Patients in the peripheral SpA (p-SpA) group were recruited based on a diagnosis of p-SpA according to the 2005 Classification and Diagnostic Criteria for Psoriatic Arthritis (CASPAR) [25], specifically selecting those with peripheral arthritis involvement. The PsO group consisted of patients with cutaneous psoriasis diagnosed by dermatologists who presented with no clinical joint symptoms. All patients in the p-SpA and PsO groups were confirmed negative for other autoimmune diseases. In the proof-of-concept preliminary study, three OA controls, three patients with PsO, and six patients with p-SpA were included for miRNA analysis.

Following the preliminary investigation, a validation cohort was established to verify the initial findings. We enrolled patients representing the broader seronegative spondyloarthritis disease spectrum (SpA-DS), which included various phenotypes such as psoriatic arthritis (PsA), ankylosing spondylitis (AS), and inflammatory bowel disease (IBD). This validation cohort was stratified into two subgroups based on clinical presentation: SpA-DS with peripheral arthritis (SpA-A) and SpA-DS without peripheral arthritis (SpA-N). The candidate miRNAs identified in the discovery phase (comparing PsO and p-SpA) were then evaluated in this cohort to determine their ability to differentiate SpA-A from SpA-N patients.

The study protocol was approved by the Institutional Review Board (IRB) of the Chang Gung Memorial Hospital in Taiwan (IRB: 104-5733B, 201700509B0, 202000775B0, 202300441B0, and 202301268B0). All the participants provided written informed consent. No individual personal data were included in any form in this study, and all the participants provided informed consent.

### 2.2. Data Collection

The demographic and clinical characteristics of the patients were analyzed, including age, leukocyte differential count, hemoglobin, hematocrit, platelet count, creatinine, uric acid, C-reactive protein, erythrocyte sediment rate, lipid levels, and comorbidities (diabetes, hypertension, gout, ankylosing spondylitis, asthma, bronchitis, and allergic dermatitis).

### 2.3. MiRNA Preparation

Blood samples were centrifuged at 1000× *g* for 10 min to pellet cellular debris. The resulting supernatants were used for RNA extraction. Total RNA was extracted from 300 µL of fluid using the miRNeasy kit (Qiagen, Germantown, MD, USA), as described in a previous study [26], followed by amplification according to the manufacturer’s instructions (Qiagen). The quality and quantity of the extracted RNA were assessed using an Agilent 2100 Bioanalyzer and NanoDrop 1000 spectrophotometer (Thermo Scientific, Waltham, MA, USA ). For the bioanalyzer, PanelChip™ (QuarkBio, Taipei, Taiwan) qPCR on PanelStation™ (QuarkBio, Taipei, Taiwan) was used for quantification and initial quality measurement, and the Small RNA chip was utilized to gain a more detailed view of RNAs in the 6- to 150-nucleotide size range. Quantitative real-time PCR (qPCR) was performed according to the manufacturer’s instructions (Qiagen MD, USA) to profile the miRNA distribution in body fluid samples. For the discovery assay, cDNA was produced using the miScript Reverse Transcription kit (Qiagen MD, USA). The authors used the Matrix Hydra eDrop (Thermo Scientific MA, USA) to mix the cDNA sample and the qPCR master reagent Human miScript Assay 384 set v10.1 (Qiagen MD, USA) to reduce pipetting error. Wells with multiple melting temperatures were excluded from further analyses. To maximize the efficiency of the initial broad screening across 149 miRNAs within small discovery cohorts, a strategic biological pooling approach was implemented, wherein 5 µL (equal volumes) of extracted total RNA from subjects within identical clinical subgroups were pooled into representative biological replicates. Methodologically, this pooling protocol serves two vital academic purposes: first, it effectively mitigates individual confounding biological variance (noise) inherent in small-sample human plasma screenings; second, it circumvents the absolute sample volume constraints regarding low-abundance total RNA yields isolated from specialized clinical sub-fractions. To eliminate any potential masking effects caused by pooling, all candidate leads tracking from this discovery engine were meticulously verified using individual, non-pooled patient samples during the subsequent independent validation phase. The miRNAs were polyadenylated and converted into cDNA using QB-universal primer reverse transcription in a single step. The qPCR reaction mixture, containing cDNA and SYBR^®^ Green Master Mix (Thermo Scientific MA, USA), was loaded onto a chip array. Red blood cell contamination and subsequent sample hemolysis represent well-documented confounders capable of falsely elevating specific circulating miRNA concentrations in blood-derived profiles. In this study, hemolysis was tightly monitored and quantitatively controlled using a dual-layered verification system. First, for miRNA-based quality indexing, miRNA arrays routinely incorporated target assessments for hsa-miR-451a (highly enriched in erythrocytes) and hsa-miR-23a-3p (stably expressed in baseline plasma). The mathematical delta-Ct threshold (∆Ct [hsa-miR-23a-3p − hsa-miR-451a]) was utilized as a standard quantitative metric to screen out hemolyzed samples prior to downstream pathway or diagnostic modeling. Second, regarding clinical covariate correlation, clinical hematocrit (Hct) and hemoglobin (Hb) concentrations were systematically documented for all study participants. Cross-group continuous variable profiles were verified using the Kruskal–Wallis test, confirming that variations in baseline systemic blood cell counts did not exert a confounding influence on measured plasma miRNA expression levels (*p* ≥ 0.05). RT spike-in RNA was used as the control for reverse transcription. qPCR spike-in DNA was used as the control for qPCR. The control assay was evaluated, and relevant data were analyzed accordingly. In this study, we detected 149 different miRNAs in the plasma samples of the patients. In the validation cohort study, we determined the miRNA concentration using TaqMan quantitative PCR. We examined each miRNA in 30 patients in the validation cohort. The miRNAs used were hsa-miR-140-5p, hsa-let-7c-5p, hsa-miR-1-3p, hsa-miR-122-5p, hsa-miR-134-5p, hsa-miR-192-5p, hsa-miR-361-5p, hsa-miR-409-3p, hsa-miR-375-3p, hsa-miR-146a-5p, hsa-miR-193b-3p, hsa-miR-125a-5p, and hsa-miR-221-3p.

### 2.4. Statistical Analysis

Patient characteristics were summarized using descriptive statistics. Normally distributed continuous variables were expressed as mean ± standard deviation (SD), whereas non-normally distributed variables were presented as the median and inter-quartile range (IQR). Categorical variables were summarized as frequencies and per-centages.

Plasma miRNA expression levels were initially compared among the three study groups using the Kruskal–Wallis test. The resulting *p*-values obtained from the 149 screened miRNAs were adjusted for multiple testing using the Benjamini–Hochberg (BH) false discovery rate (FDR) procedure. Candidate miRNAs with an adjusted q-value (FDR) < 0.05 were considered statistically significant and selected for further analysis. For these significant miRNAs, post hoc pairwise comparisons were subsequently performed using Dunn’s test with Bonferroni adjustment to identify the specific group differences (p-SpA vs. PsO, p-SpA vs. OA, and PsO vs. OA).

Multivariate analysis was performed using logistic regression to estimate odds ratios (ORs) and 95% confidence intervals (CIs). The diagnostic performance of individual miRNAs was evaluated using receiver operating characteristic (ROC) curve analysis, and the area under the curve (AUC) with corresponding 95% confidence intervals and significance levels was reported. In addition, composite miRNA models were explored using multivariable logistic regression to evaluate the combined diagnostic performance of selected candidate miRNAs. Categorical variables were compared using the chi-square test, including analyses of comorbidity prevalence between patients with psoriasis without clinical arthritis (PsO) and those with psoriatic spondyloarthritis (p-SpA).

Sample size estimation was based on a two-sided one-way ANOVA pairwise comparison. Assuming a mean Ct difference of 1.0 with a standard deviation of 1.0, a minimum of eight samples per comparison was estimated to provide 80% statistical power. Therefore, the initial discovery cohort (n = 12; three PsO, six p-SpA, and three OA controls) was designed as an exploratory, hypothesis-generating screening cohort rather than a definitive statistical validation cohort.

During the discovery phase, expression profiling of 149 plasma miRNAs was performed using the QuarkBio PanelStation™ platform. Each miRNA was initially evaluated using the Kruskal–Wallis test across the three study groups. The resulting *p*-values were adjusted using the Benjamini–Hochberg FDR procedure to control for multiple testing across all 149 miRNAs. Candidate miRNAs with an adjusted q-value (FDR) < 0.05 were selected for further evaluation. For these significant candidates, Dunn’s post hoc test with Bonferroni adjustment was subsequently performed to determine which pairwise group comparisons accounted for the overall differences.

The selected candidate miRNAs were independently evaluated in the validation cohort (n = 30) using single-assay TaqMan quantitative PCR. Statistical analyses in the validation cohort included comparisons of miRNA expression among study groups using the Kruskal–Wallis test followed by Dunn’s post hoc test with Bonferroni adjustment where appropriate. Statistical significance in the validation cohort was defined as *p* < 0.05. The robustness and clinical utility of the validated miRNAs were further assessed using receiver operating characteristic (ROC) curve analysis, area under the curve (AUC), and multivariable logistic regression models.

### 2.5. Pathway Analysis

Raw quantitative real-time PCR (Ct) data were standardized utilizing a robust normalization matrix implemented strictly according to the manufacturer’s technical specifications (Quark Biosciences Inc., Taipei, Taiwan). To ensure empirical stability across array chips, the mathematical transformation integrated both endogenous controls and standardized baseline reference values derived from the global mean expression profile of the 149-miRNA panel. For the single-target TaqMan validation assays, absolute data stability was controlled, and uniform inputs were maintained across all validation templates to ensure cross-batch reproducibility. Fold changes and *p*-values were calculated relative to OA controls to identify miRNAs significantly expressed in the p-SpA and PsO groups. To isolate disease-specific signatures, miRNAs significantly expressed in the PsO group were excluded from the p-SpA profile. This comparative approach enabled the identification of significant miRNAs unique to p-SpA while filtering out background signals associated with OA controls. The remaining miRNAs were further analyzed to identify potential downstream target genes and associated immune pathways using the Kyoto Encyclopedia of Genes and Genomes (KEGG) database. We utilized the online databases DIANA tools TarBase v.8 [27] and mirPath v.3 [28] for in-depth analysis of miRNA-related pathways and heatmap visualization.

## 3. Results

### 3.1. Demographic Characteristics of Patients

A total of 12 subjects—comprising six with p-SpA, three with PsO, and three osteoarthritis (OA) controls—were recruited for this preliminary study. No significant differences were observed among the subgroups regarding total leukocyte counts, platelet counts, hemoglobin, hematocrit, inflammatory markers (C-reactive protein and erythrocyte sedimentation rate), or levels of AST, ALT, and rheumatoid factor (all *p* ≥ 0.05; Table 1). For the validation phase, a validation cohort of 30 patients was enrolled to measure and confirm miRNA expression levels.

### 3.2. Results of PanelChip™ qPCR on QuarkBio PanelStation™ and Comparison Between p-SpA and PsO Patients

The results of PanelChip™ qPCR on the QuarkBio PanelStation™ are shown in Supplementary Table S1. The distribution patterns of the different diseases were compared with those of the OA control. Diseases, including PsO and p-SpA, are shown, and the resulting clusters of all samples were based on miRNA expression patterns (Supplementary Table S1).

### 3.3. KEGG Pathway Analysis and Comparison Between p-SpA and PsO Patients

**Figure 1:**
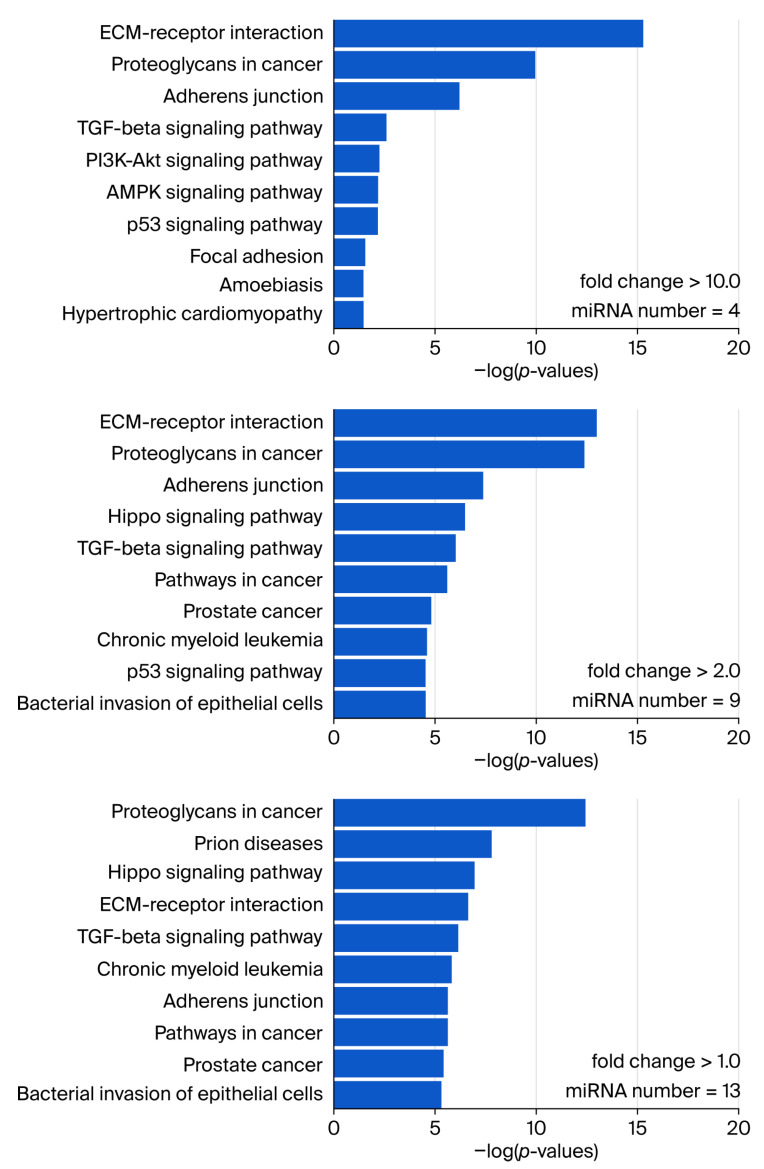
KEGG pathway enrichment analysis of differentially expressed circulating miRNAs between the p-SpA and PsO groups in the preliminary discovery phase (n = 9). The bar charts indicate the statistical significance (−log(*p*-value)) of altered gene signaling networks regulated by selected miRNAs across different expression thresholds: fold change > 10.0 involving 4 miRNAs (**upper**), fold change > 2.0 involving 9 miRNAs (**middle**), and fold change > 1.0 involving 13 miRNAs (**lower**), analyzed according to KEGG Orthology.

Comparisons were performed among the three subgroups (PsO, p-SpA, and OA controls), and miRNAs showing significant differential expression between the PsO and p-SpA groups were selected for further analysis (Table 2). Due to the exploratory nature and limited sample size of this initial discovery phase, the subsequent pathway identifications are considered strictly hypothesis-generating. Potential immune pathways identified through KEGG analysis included extracellular matrix (ECM)/receptor interaction (hsa04512) and proteoglycans in cancer (hsa05205), which were the most significantly enriched pathways. Proteoglycans potentially involved in the psoriasis-related SpA spectrum include hyaluronate, heparan sulfate proteoglycan, chondroitin sulfate, and keratan sulfate (Figure 1). Furthermore, ECM–receptor interactions were associated with the bioinformatically identified activation of several mesenchymal genes, such as fibronectin 1; collagen types I, III, and IV; and integrin alpha and beta genes.

### 3.4. Involvement of Immune Regulation/Reaction in PsO Patients

We focused on KEGG pathways related to immune regulation based on the target genes of the differentially expressed miRNAs. Rather than listing all altered cascades, we highlighted the most biologically relevant networks distinguishing the p-SpA and PsO groups. These predominantly included innate immune responses—such as the Toll-like receptor (TLR) signaling pathway (hsa04620), neutrophil and myeloid cell differentiation (hsa04640), and antigen processing through the MHC class I pathway (hsa04612)—as well as adaptive immune cascades, most notably Th17 (hsa04659) and Th2 (hsa04658) cell differentiation pathways.

### 3.5. Validation of the MiRNA Levels in SpA-A and SpA-N Patients

**Figure 2:**
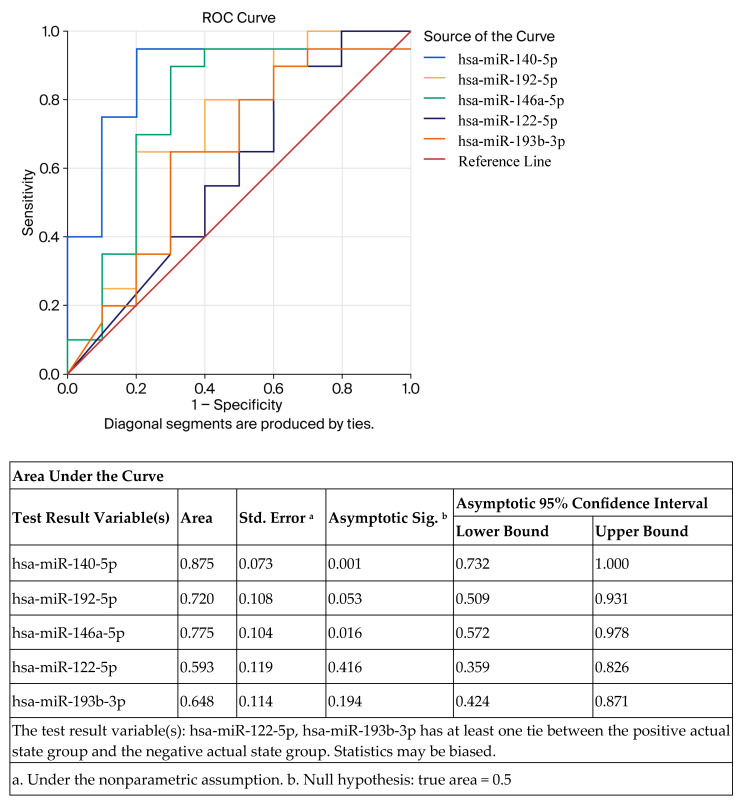
Receiver operating characteristic (ROC) curve analysis of candidate circulating miRNAs for discriminating SpA-A from SpA-N patients in the validation cohort. hsa-miR-140-5p showed the highest discriminatory performance, with an AUC of 0.875 (95% CI: 0.732–1.000, *p* = 0.001), followed by hsa-miR-146a-5p (AUC = 0.775, 95% CI: 0.572–0.978, *p* = 0.016) and hsa-miR-192-5p (AUC = 0.720, 95% CI: 0.509–0.931, *p* = 0.053). AUC, area under the curve; CI, confidence interval; ROC, receiver operating characteristic; SpA-A, spondyloarthritis disease spectrum with peripheral arthritis; SpA-N, spondyloarthritis disease spectrum without peripheral arthritis.

We examined candidate miRNAs in 30 patients from the validation cohort. The miRNAs used were hsa-miR-140-5p, hsa-let-7c-5p, hsa-miR-1-3p, hsa-miR-122-5p, hsa-miR-134-5p, hsa-miR-192-5p, hsa-miR-361-5p, hsa-miR-409-3p, hsa-miR-375-3p, hsa-miR-146a-5p, hsa-miR-193b-3p, hsa-miR-125a-5p, and hsa-miR-221-3p. Among the 14 miRNAs checked in the validation cohort, three miRNAs—hsa-miR-140-5p, hsa-miR-192-5p, and hsa-miR-146a-5p—were found to be significantly differentially expressed between SpA-A and SpA-N patients (all *p* ≤ 0.05; *p* = 0.05 for hsa-miR-192-5p, Table 3). To evaluate the clinical discriminatory power of these markers, ROC analysis further confirmed their diagnostic performance. Specifically, hsa-miR-140-5p demonstrated the highest performance with an AUC of 0.875 (*p* = 0.001); at the optimal cut-off value of 36.16, it exhibited a sensitivity of 95% and a specificity of 80%. Furthermore, hsa-miR-146a-5p yielded an AUC of 0.775 (*p* = 0.016), with a sensitivity of 90% and a specificity of 70% at a cut-off of 35.41. Lastly, hsa-miR-192-5p yielded an AUC of 0.720 (*p* = 0.053), demonstrating a sensitivity of 75% and a specificity of 60% at a cut-off of 34.71 (Figure 2).

### 3.6. KEGG Pathway Convergence in the Validation Phase

**Figure 3:**
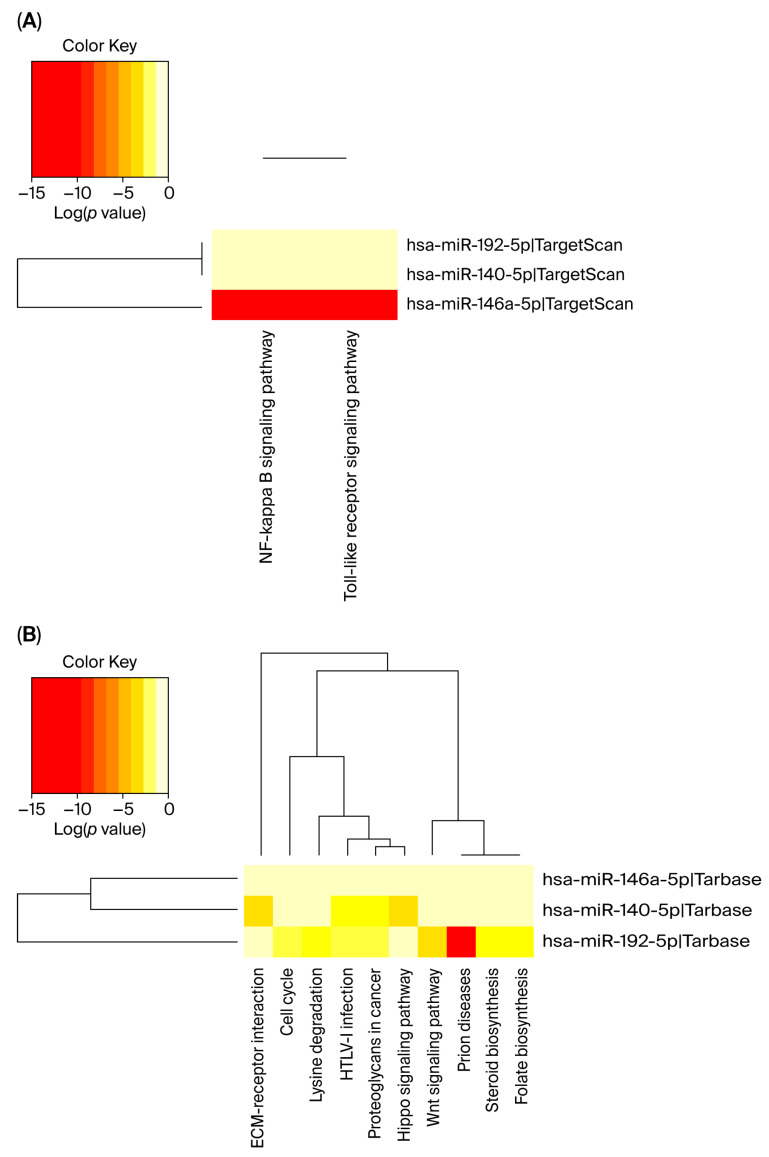
Integrated KEGG pathway enrichment analysis of the three discriminative plasma miRNAs (hsa-miR-140-5p, hsa-miR-146a-5p, and hsa-miR-192-5p) in the validation cohort (n = 30). (**A**) Heatmap depicting predicted target pathways identified via the TargetScan algorithm, highlighting the shared targeting of the NF-κB and Toll-like receptor (TLR) signaling pathways. (**B**) Heatmap depicting experimentally supported target pathways identified via the DIANA-TarBase v8.0 database, illustrating functional convergence on networks including ECM–receptor interaction, Wnt, and Hippo signaling pathways. Note: The color intensity gradient represents statistical significance based on log(*p*-value), spanning from deep red (highly significant, log(*p*) ≈ −15) to pale yellow (non-significant). Clustering dendrograms group miRNAs (rows) and pathways (columns) based on the similarity of their enrichment profiles.

To explore potential functional pathways associated with peripheral arthritis, KEGG pathway enrichment analyses were performed on the targets of the three validated miRNAs (hsa-miR-140-5p, hsa-miR-146a-5p, and hsa-miR-192-5p). The integrated analysis revealed a distinct dual-layered molecular regulation pattern (Figure 3). Computational prediction via the TargetScan [28] algorithm demonstrated a clear functional convergence, with the NF-κB signaling pathway (hsa04064) and Toll-like receptor (TLR) signaling pathway (hsa04620) identified as the primary inflammatory cascades simultaneously targeted by all three miRNAs (Figure 3A). Conversely, screening against the experimentally validated TarBase v8.0 [27] database highlighted significant enrichment in structural and homeostatic networks, specifically ECM–receptor interaction (hsa04512), Wnt signaling (hsa04310), and Hippo signaling (hsa04390) (Figure 3B). Downstream mapping suggested that these immune- and bone-remodeling-related cascades may be linked to potential mediators, including *CXCL2*, *TRAF6*, and *IRAK1*. (Additional database permutations are provided in Supplementary Figure S1).

## 4. Discussion

This study integrates a preliminary discovery phase with a validation cohort, yielding several key findings that provide a comprehensive overview of the molecular differences between PsO and p-SpA, as well as the distinct profiles between the SpA-A and SpA-N groups. However, we continuously emphasize that the initial discovery screening was strictly exploratory. Any interpretations regarding the clinical relevance and diagnostic potential of these early miRNA signatures must be viewed as hypothesis-generating observations that require extensive validation.

Future studies involving larger cohorts will be conducted to refine our findings regarding specific pathways and the potential mechanisms underlying pathogenesis. In the current study, we primarily enrolled male patients to minimize the influence of hormones on miRNA expression and to reduce potential confounding from seronegative rheumatoid arthritis. Comparison of plasma miRNA profiles revealed that patients with PsO exhibited a higher number of enriched pathways in their circulation. Key enriched pathways predominantly involved critical innate and adaptive immune cascades, including antigen processing and presentation through the MHC class I pathway, neutrophil and myeloid cell differentiation, and Th17 cell differentiation. Most notably, the Toll-like receptor (TLR) signaling pathway emerged as a primary candidate, which was subsequently validated in our validation cohort. These findings provide a proof of concept that patients initially without clinical joint symptoms can already manifest systemic immune pathway activation, suggesting that overt p-SpA represents a distinct clinical phenotype within the same spondyloarthritis spectrum. This is consistent with clinical observations that approximately 30% of patients with psoriasis may eventually develop overt p-SpA.

For the validation cohort, we enrolled a broad spectrum of patients with seronegative spondyloarthritis—including those with p-SpA, PsO, AS, and IBD—to minimize potential selection bias. Participants were subsequently stratified into two subgroups: those with arthritis (SpA-A) and those without (SpA-N). We chose to include a broad spectrum of SpA-DS phenotypes because we hypothesize that the molecular pathogenesis of peripheral arthritis is distinct from purely axial involvement, regardless of specific extra-articular manifestations. For instance, psoriatic arthritis (PsA), ankylosing spondylitis (AS), and IBD-related spondyloarthritis all encompass both axial and peripheral subtypes. By grouping these conditions based on the presence (SpA-A) or absence (SpA-N) of peripheral involvement, we aimed to maximize the miRNA feasibility of discriminating peripheral arthritis across the entire SpA spectrum. We validated the candidate miRNAs identified in the discovery phase, utilizing one miRNA as a positive control [29,30] and two others as negative controls. The results indicated that hsa-miR-140-5p, hsa-miR-192-5p, and hsa-miR-146a-5p were significantly differentially expressed between the SpA-A and SpA-N groups (all *p* ≤ 0.05; Table 3). Notably, hsa-miR-140-5p, which was first identified in our discovery phase, remained a highly significant discriminator in this larger cohort. Our multivariable logistic regression models demonstrated that hsa-miR-140-5p remains a robust independent discriminator even after accounting for systemic inflammatory markers such as CRP. The preliminary efforts to integrate these markers into a composite miRNA score suggest a synergistic effect in identifying peripheral involvement. Furthermore, the positive control hsa-miR-146a-5p—consistent with previous research—demonstrated a robust ability to distinguish between SpA-A and SpA-N phenotypes.

Delayed diagnosis of arthritis in patients with psoriasis not only postpones essential treatment but also increases the risk of irreversible joint damage [31]. Underdiagnosis of psoriatic disease remains a significant concern, with one study reporting a prevalence of approximately 9% [32]; this issue is equally pertinent to patients within the seronegative spondyloarthritis spectrum. Such joint damage may be attributed to the prolonged activation of potential pathways, including the Th17 cell differentiation pathway, MHC class I pathway, neutrophil and myeloid cell differentiation pathway, NK cell activation pathway, or even the FcγRI signaling pathway. Although the clinically available IL-17 antagonist is a useful treatment against p-SpA, anti-tumor necrosis factor alpha remains a key alternative for patients with arthritis across the spondyloarthritis (SpA) spectrum [33,34,35,36]. This clinical distinction is supported by our pathway analysis, which identified the enrichment of TLR and NF-κB multi-inflammatory signaling specifically in SpA-A patients, rather than in SpA-N.

Based on our bioinformatic predictions, we hypothesize that the TLR and NF-κB multi-inflammatory pathways, mediated by computationally identified key genes (*TRAF6*, *IRAK1*, and *CXCL2*), may be involved in distinguishing SpA-A from SpA-N. The heatmap (Figure 3) illustrates the functional convergence among hsa-miR-140-5p, hsa-miR-146a-5p, and hsa-miR-192-5p in regulating these immune networks. The use of the TarBase v8.0 database provides database-supported evidence for these enriched networks and suggests a potential cooperative involvement of these miRNAs in immune responses within the SpA spectrum.

Consistent with recently published genetic risk analyses of p-SpA [18], both the existing literature and our current findings identify NF-κB signaling as a critical discriminator between the p-SpA and PsO phenotypes. While NF-κB drives chronic inflammation, enthesitis, and irreversible joint damage in p-SpA, hsa-miR-146a-5p functions as a key negative regulator—potentially by targeting IRAK1 and TRAF6. This suggests that miRNA-mediated modulation may influence the clinical expression of genetic risk. Such convergence further reinforces the role of NF-κB as a pivotal therapeutic target, supporting the use of biologics such as TNF-α inhibitors in these patients.

Hsa-miR-140-5p has demonstrated significant clinical relevance in systemic inflammatory diseases [37,38,39,40]. Mechanistically, hsa-miR-140-5p targets key effectors involved in YAP/TAZ crosstalk via Hippo/Wnt integration, a pathway particularly relevant to the pathological bone involvement in the spondyloarthritis spectrum. Our findings suggest that miRNA/Wnt interactions may serve as potential biomarkers to discriminate p-SpA phenotypes from PsO (psoriasis without clinical arthritis). Notably, in our preliminary discovery phase, hsa-miR-140-5p was the sole miRNA that showed potential to differentiate among the p-SpA, PsO, and OA control groups (Table 2); nevertheless, this observation must be interpreted with caution due to the inherent false-positive risks of the small initial sample size.

Regarding hsa-miR-192-5p, its altered levels in the mucosa of patients with Crohn’s disease [41] support its link to intrinsic cellular inflammatory mechanisms. This finding reflects a potential universal inflammatory pathway underlying the broader SpA spectrum, consistent with the discriminative role of hsa-miR-192-5p observed in our validation cohort.

Despite the promising diagnostic potential of these circulating miRNAs, it is crucial to distinguish between this preliminary discriminative performance and actual clinical implementation. Importantly, these proposed biomarkers have not yet undergone external validation in a completely independent population. Furthermore, several translational hurdles must be addressed before their implementation into routine clinical practice. First, a major technical barrier is the lack of standardized protocols for plasma miRNA isolation, quantification, and data normalization across different clinical laboratories, which currently complicates the establishment of universally defined diagnostic cut-off thresholds. Second, economic factors present a significant challenge; the current cost of specialized quantitative PCR assays remains substantially higher than that of conventional, widely accessible inflammatory markers such as CRP or ESR. Furthermore, the clinical adoption of miRNA profiling necessitates specialized technical expertise and rigorous training for medical technologists to ensure assay reproducibility and to minimize confounding variables, such as sample hemolysis or cross-contamination. Addressing these technical, financial, and logistical barriers, along with rigorous external validation through large-scale multi-center trials, will be essential to successfully transition these miRNA panels from bench to bedside.

Our study has several limitations that warrant consideration. First, certain clinical data were missing, some miRNAs were below the limit of detection, and the absence of longitudinal data precluded follow-up assessments or the evaluation of temporal changes. Second, the small sample size of the discovery cohort (n = 12) is a significant limitation. Although this initial phase provided a basis for identifying candidates, such a small cohort increases the risk of false positives and potential overfitting. Accordingly, the present study is intended to be exploratory and hypothesis-generating in nature. The identified miRNA signatures serve as a foundation for future larger-scale trials to confirm their diagnostic utility. Third, the cross-sectional design of this study limits our ability to establish definitive causal relationships. Finally, several potentially relevant confounders and lifestyle variables were not available in our dataset, including smoking status [42], co-existing inflammatory bowel disease [43], alcohol consumption [44,45,46,47], body mass index (BMI), total disease duration, and detailed treatment exposure. Because these variables are known to potentially influence circulating miRNA levels and the overall inflammatory profile, their absence represents a limitation of the current study. Future studies should incorporate these clinical covariates into multivariable models to further refine the discriminative accuracy of the miRNA signatures. Furthermore, the composition of our validation cohort introduces considerable biological heterogeneity by pooling distinct SpA phenotypes (such as PsA, AS, and IBD-related SpA) and stratifying them solely by the presence or absence of peripheral arthritis. While this approach reflects real-world clinical attempts to identify a universal peripheral biomarker, we must acknowledge the possibility that the observed miRNA profiles could partially reflect inherent molecular differences among these underlying disease subsets, rather than the peripheral arthritis pathogenesis itself. Future studies must validate these findings in larger, disease-specific subgroups.

In conclusion, the intersection of the NF-κB and Wnt signaling pathways provides hypothesis-generating evidence for their potential involvement in SpA-A-associated molecular profiles, effectively distinguishing it from the SpA-N phenotype. These shared molecular themes support an integrated model: hsa-miR-146a-5p may mitigate NF-κB-mediated genetic risk, while hsa-miR-140-5p influences Wnt-mediated pathological bone remodeling. Our findings indicate that arthritis in the spondyloarthritis spectrum is characterized by the activation of multiple immune pathways regulated by specific plasma miRNAs (hsa-miR-140-5p, hsa-miR-192-5p, and hsa-miR-146a-5p) and their functional gene targets (*TRAF6*, *IRAK1*, and *CXCL2*). This distinct molecular signature underscores the necessity for earlier and more intensive therapeutic interventions in patients presenting with the SpA-A phenotype.

## 5. Conclusions

Regarding immune regulation and reaction-associated pathways, our data demonstrate that both innate and adaptive immunity are differentially activated between patients with PsO and those with p-SpA. Overall, the NF-κB signaling pathway (hsa04064) and the Toll-like receptor (TLR) signaling pathway (hsa04620) emerged as the two most significant pathways distinguishing the SpA-A and SpA-N phenotypes. This potential differential activation is associated with distinct profiles of circulating plasma miRNAs (hsa-miR-140-5p, hsa-miR-192-5p, and hsa-miR-146a-5p). While enrichment analyses computationally predict the involvement of key target genes (*TRAF6*, *IRAK1*, and *CXCL2*), these findings are strictly hypothesis-generating. Future experimental studies providing direct functional validation are necessary to confirm these mechanistic observations.

## Figures and Tables

**Table 1:** Demographic data of preliminary study patients in the preliminary study (N = 12).

	Osteoarthritis (n = 3)	p-SpA (n = 6)	PsO (n = 3)	*p*-Value *
Age (years)	50 (40, 60)	45 (37.5, 52.5)	43.33 (38, 48)	0.576
Leukocytes (1000/μL)	5766 (5000, 6800)	7016 (4950, 8225)	4900 (4100, 6400)	0.516
Neutrophils (%)	66.73 (65, 69.2)	64.8 (58.8, 70.9)	65.56 (60, 72.3)	0.751
Lymphocytes (%)	25.7 (22.1, 30)	29.58 (23.7, 36.3)	25.7 (20, 31.4)	0.931
Monocytes (%)	6.066 (4.5, 8.7)	5.616 (4.55, 7.05)	8.733 (7.7, 9.9)	0.143
Platelets (1000/μL)	229.3 (215, 253)	290.8 (236, 347)	191.3 (160, 245)	0.293
Hemoglobin (g/dL)	14.36 (13, 15.3)	13.66 (11.9, 15.4)	12.5 (10.1, 13.8)	0.146
Hematocrit (%)	46.8 (43.9, 50)	39.8 (34.7, 45.4)	37.7 (30.8, 41.4)	0.122
Creatinine (mg/dL)	0.8 (0.7, 0.9)	1.03 (0.67, 1.15)	0.806 (0.63, 0.99)	0.612
Uric acid (mg/dL)	4.933 (3.5, 6)	7.05 (5.12, 7.1)	8.33 (8, 9)	0.048
C-reactive protein (mg/L)	9.376 (0.83, 24.3)	3.52 (1.04, 23.5)	4.556 (0.42, 12.1)	0.881
Erythrocyte sedimentation rate (mm/h)	5.666 (3, 9)	5 (2, 9.25)	4 ± 2	1.000
Diabetes	0	0	0	X
Hypertension	0	0	0	X
Gout	0	0	0	X
Ankylosing spondylitis	0	0	0	X
Asthma	0	0	0	X
Bronchitis	0	0	0	X
Allergic dermatitis	0	0	0	X

* Comparison between the three groups: OA, p-SpA, and PsO subgroups. X, not assessed. All expressed as median (25% and 75% interquartile values); *p*-value is calculated with Kruskal–Wallis test.

**Table 2:** Chosen miRNAs expressed differently between PsO and p-SpA patients after normalization with OA controls in the preliminary study (all *p*-values < 0.05).

miRID	p-SpA Ct	PsO Ct	OA Ct	*p*-Value
				p-SpA vs. PsO
^§^ hsa-miR-140-5p	28.57 ± 0.23	29.84 ± 0.84	31.48 ± 0.54	0.05
^¥^ hsa-let-7c-5p	28.85 ± 3.49	29.21 ± 1.17	32.58 ± 2.20	0.02
^¥^ hsa-miR-1-3p	29.53 ± 1.23	31.30 ± 0.87	31.31 ± 1.07	0.03
^¥^ hsa-miR-122-5p	27.16 ± 1.94	30.01 ± 1.48	28.68 ± 0.11	0.02
^¥^ hsa-miR-134-5p	28.26 ± 0.46	28.62 ± 0.00	28.31 ± 0	0.01
^¥^ hsa-miR-192-5p	28.41 ± 0.74	29.18 ± 0.56	28.93 ± 0.58	0.02
^¥^ hsa-miR-1972	26.56 ± 2.83	25.44 ± 0.55	25.93 ± 0.65	0.03
^¥^ hsa-miR-361-5p	29.11 ± 2.77	28.09 ± 0.92	30.99 ± 1.17	0.02
^¥^ hsa-miR-409-3p	27.65 ± 0.94	29.60 ± 1.46	32.85 ± 0	0.01
^¥^ hsa-miR-375	32.66 ± 0.48	30.39 ± 0.31	31.99 ± 0	0.01

p-SpA, peripheral spondyloarthritis; PsO, psoriasis without clinical arthritis; OA, osteoarthritis. Fold change demonstrates only absolute numbers. ^§^ Direct comparison between p-SpA, PsO, and OA with Kruskal–Wallis test; ^¥^ indirect comparison between p-SpA and PsO with Mann–Whitney U Test.

**Table 3:** Demographic and clinical characteristics of patients in the validation cohort (N = 30).

N = 30	SpA-A	SpA-N	*p*-Value
Number	20	10	ND
Age (years)	43.5 (35, 55.5)	41.25 (31.75, 52.8)	0.69
Leukocytes (1000/μL)	6.05 (5.025, 8.075)	6.5 (4.85, 7.9725)	0.96
Neutrophils (%)	59.6 (51.825, 70.375)	69.85 (57.5, 75.425)	0.12
Lymphocytes (%)	31.4 (22.675, 38.575)	22.15 (18.225, 32.25)	0.16
Monocytes (%)	5.7 (4.775, 7.1)	6 (5.1, 7)	0.72
Eosinophil (%)	2.3 (1.825, 3.925)	1.55 (0.75, 2.9)	0.21
Basophil (%)	0.5 (0.275, 0.8)	0.4 (0.2, 0.625)	0.36
Platelets (1000/μL)	250.5 (213.25, 288)	324 (235.5, 394)	0.03 *
Hemoglobin (g/dL)	14.55 (12.675, 15.3)	12.7 (12, 15)	0.28
Hematocrit (%)	43.4 (37.8, 45.325)	39.7 (35.55, 43.1)	0.15
Erythrocyte sedimentation rate (mm/h)	7.5 (5.25, 12)	9 (3, 26.5)	0.51
Total cholesterol	193.5 (151.25, 209.5)	228 (101.5, 234)	0.49
Triglyceride	140 (92.75, 184.75)	109 (81, 253.25)	0.75
High-density lipoprotein	46 (35.5, 56.75)	39 (20.75, 67.75)	0.60
AC sugar	99 (87, 108)	126 (95, 145)	0.15
HbA1c	6 (5.225, 6.375)	5.55 (5.275, 7.25)	0.83
Aspartate aminotransferase (mg/dL)	26.5 (19, 29.75)	21 (18.25, 31)	0.53
Alanine aminotransferase (mg/dL)	27 (18.5, 46.5)	17.5 (12, 31.25)	0.10
Creatinine (mg/dL)	0.855 (0.7025, 0.9875)	0.83 (0.6, 1.015)	0.68
Uric acid (mg/dL)	6.05 (4.75, 6.9)	5.2 (3.8, 5.8)	0.17
C-reactive protein (mg/L)	1.65 (0.5975, 2.7975)	1.75 (0.86, 3.9475)	0.48
Total IgE	25.1 (8.32, 77.075)	11.8 (11.8, 11.8)	0.70
Eosinophil cationic protein	9.37 (4.83, 19.5)	50 (50, 50)	0.13
hsa-miR-140-5p	35.89 (33.01, 36.42)	38.69 (37.63, 39.59)	<0.01 *
hsa-miR-122-5p	37.39 (33.34, 40.00)	38.50 (35.20, 40.00)	0.41
hsa-miR-192-5p	34.51 (32.47, 36.34)	36.99 (34.71, 38.25)	0.05
hsa-miR-146a-5p	34.53 (30.46, 36.33)	36.58 (35.82, 38.24)	0.02 *
hsa-miR-193b-3p	37.86 (36.32, 39.32)	38.76 (37.42, 39.47)	0.19

* *p* < 0.05; ND, not determined; SpA-A, spondyloarthritis disease spectrum with peripheral arthritis; SpA-N, spondyloarthritis disease spectrum with no peripheral arthritis.

## Data Availability

All the data are available on request.

## Supplementary Materials

The following supporting information can be downloaded at https://www.mdpi.com/article/10.3390/medicina62071314/s1. Figure S1: Permutation analyses of KEGG pathway enrichment using distinct combinations of computational predictions (TargetScan) and experimentally validated (TarBase v8.0) target databases. Table S1: The 149 miRNA levels (mean ± standard deviation) from three patient subgroups in the preliminary study.
