# Supplementary material for: Potential of Circulating MicroRNA Panels to Discriminate Peripheral Arthritis in the Spondyloarthritis Spectrum: A Preliminary and Validation Study
Source: Medicina (Kaunas). 2026 Jul 8;62(7):1314. doi: 10.3390/medicina62071314 (PMC13413518; doi:10.3390/medicina62071314)
Supplement: Supplementary file 1 [file medicina-62-01314-s001.zip › Supplementary Figure S1.pdf]

Figure (A)

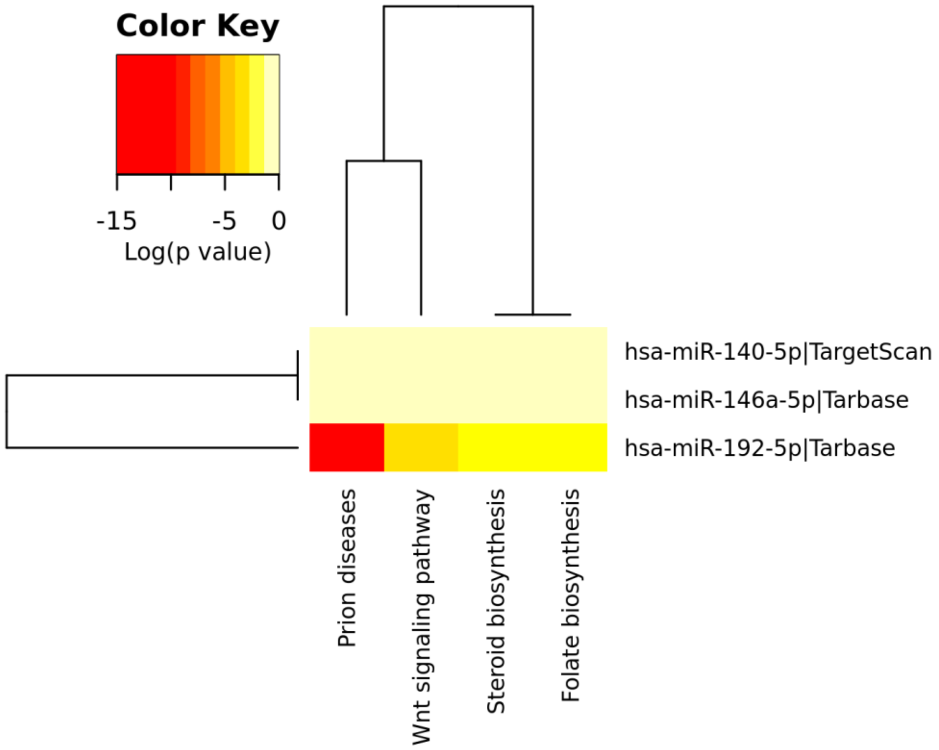

Figure (B)

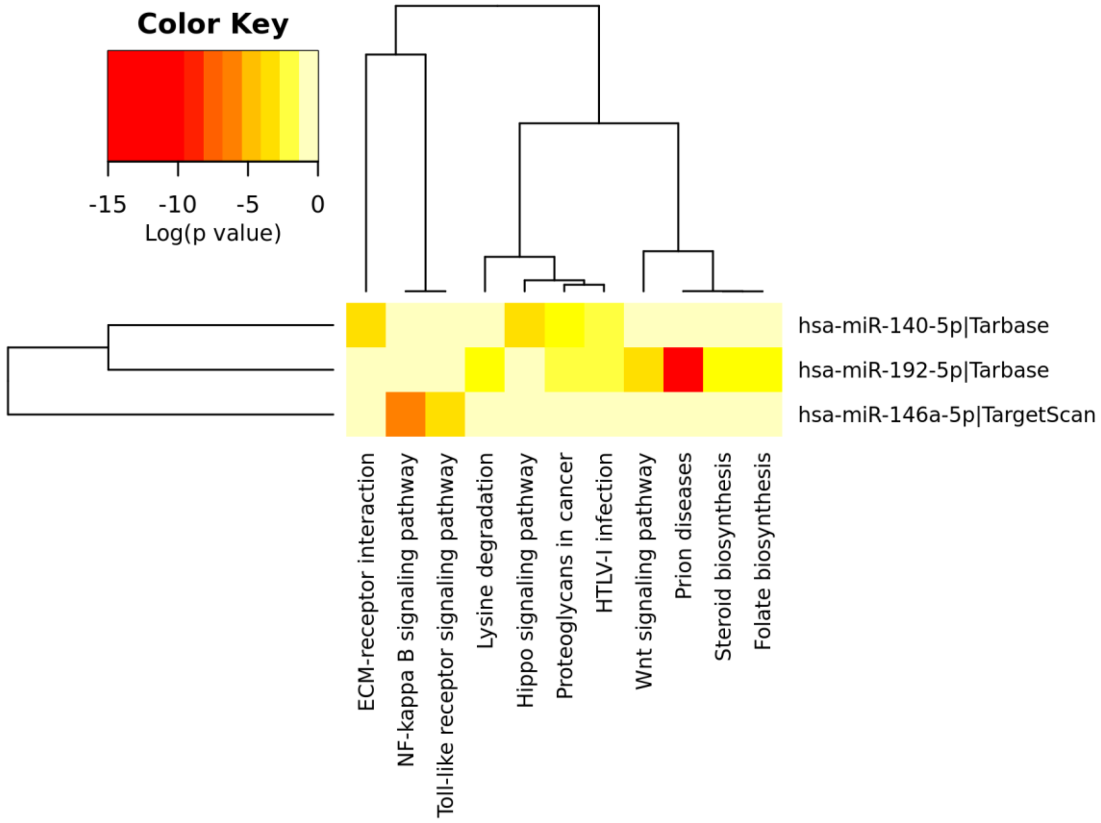

Figure (C)

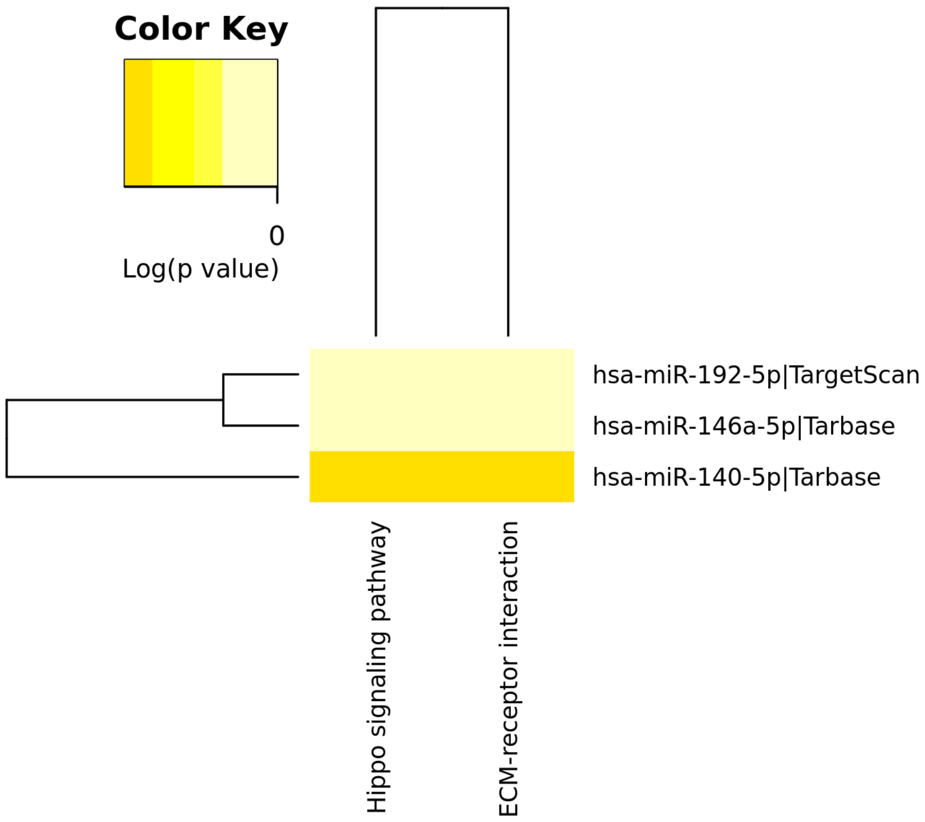

Figure S1. Permutation analyses of KEGG pathway enrichment using distinct combinations of computational predictions (TargetScan) and experimentally validated (TarBase v8.0) target databases.

(A) Permutation Phase I (TargetScan-derived hsa-miR-140-5p mixed with TarBase-validated hsa-miR-146a-5p and hsa-miR-192-5p): This heatmap illustrates the coordinated enrichment profile when analyzing the predicted targets of hsa-miR-140-5p alongside the experimentally verified targets of hsa-miR-146a-5p and hsa-miR-192-5p, clustering pathways such as Wnt signaling and steroid/folate biosynthesis.

(B) Permutation Phase II (TargetScan-derived hsa-miR-146a-5p mixed with TarBase-validated hsa-miR-140-5p and hsa-miR-192-5p): This panel maps the expanded biological network generated by combining the predicted targets of the inflammatory regulator hsa-miR-146a-5p with the experimentally supported targets of hsa-miR-140-5p and hsa-miR-192-5p, showing a broader signaling cross-talk across ECM-receptor interaction, NF-kB, Toll-like receptor, Hippo, and Wnt signaling pathways.

(C) Permutation Phase III (TargetScan-derived hsa-miR-192-5p mixed with TarBase-validated hsa-miR-146a-5p and hsa-miR-140-5p): This profile depicts the targeted convergence on Hippo signaling and ECM-receptor interaction pathways when integrating the computationally predicted targets of hsa-miR-192-5p with the experimentally validated interactions of hsa-miR-146a-5p and hsa-miR-140-5p.

Note: For all panels, the color intensity gradient represents statistical significance based on log(p-value), spanning from deep red (highly significant, log(p) ≈ -15) to pale yellow (non-significant). Key pathways are hierarchically clustered and ordered by decreasing statistical significance.
