# Supplementary material for: Potential of Circulating MicroRNA Panels to Discriminate Peripheral Arthritis in the Spondyloarthritis Spectrum: A Preliminary and Validation Study
Source: Medicina (Kaunas). 2026 Jul 8;62(7):1314. doi: 10.3390/medicina62071314 (PMC13413518; doi:10.3390/medicina62071314)
Supplement: Supplementary file 1 [file medicina-62-01314-s001.zip › Supplementary Table S1.pdf]

Supplementary Table S1. 149 microRNA levels (mean ± standard deviation) from three subgroups patients in the preliminary study.

	let-7a-5p	miR-2114-3p	miR-20a-5p	miR-222-3p	miR-143-3p	miR-17-3p	miR-181a-5p	miR-103a-3p	miR-202-3p	let-7d-3p	miR-101-3p	miR-122-5p	miR-1254	miR-125b-5p	miR-126-3p
p-SpA	27.24±4.27	ND	28.74±1.68	26.57±2.51	28.09±2.50	31.21±0.91	26.88±3.11	24.31±3.89	33.54±1.09	29.02±3.19	25.71±3.35	27.16±1.94	31.11±1.52	30.24±3.96	25.53±4.61
PsO	25.50±0.99	ND	29.44±0.76	25.64±1.08	28.46±0.90	30.49±0.20	26.19±1.41	21.69±1.10	34.60±0	28.03±1.07	23.83±0.47	30.01±1.48	31.14±0.99	27.93±1.24	22.66±1.39
OA	30.46±2.81	ND	31.05±0.98	28.76±1.66	33.06±1.39	32.63±0	29.82±1.32	26.11±2.09	35.22±0	30.80±0.77	23.69±0.80	28.68±0.11	33.03±0	31.80±1.35	25.72±0.78

	miR-1290	miR-142-3p	miR-145-5p	miR-146a-5p	miR-150-5p	miR-151a-3p	miR-152-3p	miR-155-5p	miR-15a-5p	miR-15b-5p	miR-1225-3p	miR-181b-5p	miR-182-5p	miR-183-5p	miR-193a-3p
p-SpA	28.42±3.29	24.83±5.00	25.99±3.54	26.21±3.93	25.96±2.66	28.13±2.85	30.06±1.94	30.71±0.67	27.04±3.79	27.18±4.20	29.79±0.49	28.72±3.58	31.04±1.53	31.97±0.21	ND
PsO	25.10±1.18	21.17±1.26	25.14±0.89	24.19±1.45	26.89±0.31	28.81±2.19	29.42±0.88	31.26±0.52	24.77±1.10	24.77±0.92	30.20±0.31	27.18±1.46	32.38±0.89	ND	33.29±0
OA	26.84±0.12	24.23±0.85	28.80±0.81	27.75±1.61	27.03±0.28	32.30±0.89	31.61±0.54	31.41±0	26.78±1.09	26.47±0.69	ND	30.13±0.99	31.96±0	31.09±0	ND

	miR-1972	miR-197-3p	miR-199a-5p	miR-19a-3p	miR-19b-3p	miR-203a-3p	miR-205-5p	miR-206	miR-214-3p	miR-215-5p	miR-191-5p	miR-22-3p	miR-224-5p	miR-24-3p	miR-26a-5p
p-SpA	26.56±2.83	26.75±2.51	24.90±2.80	28.34±3.11	24.10±3.78	ND	31.99±0.43	32.49±0.87	32.14±1.05	28.58±0	24.72±4.06	24.93±4.01	28.48±0.90	24.92±4.81	24.72±4.98
PsO	25.44±0.55	27.75±2.50	25.02±1.49	26.41±0.68	21.67±0.45	ND	31.33±0.56	ND	31.75±0.49	31.81±0	22.28±1.17	21.99±0.98	29.96±1.98	21.45±1.19	21.44±1.28
OA	25.93±0.65	28.80±0.04	29.55±2.25	28.00±0.88	22.90±0.64	ND	31.32±0.00	ND	31.61±0.72		25.70±1.64	23.98±1.04	32.59±0	24.62±1.34	25.12±1.68

	miR-140-5p	miR-28-3p	miR-299-5p	miR-29a-5p	miR-23a-3p	miR-423-3p	miR-31-5p	miR-129-5p	miR-326	miR-335-5p	miR-338-5p	miR-361-5p	miR-372-3p	miR-451a	miR-423-5p
p-SpA	28.57±0.23	29.72±1.76	31.08±0.47	31.11±0.38	23.66±4.10	25.75±2.63	33.08±0	30.56±0.06	25.85±0.79	27.51±0.82	33.24±0.91	29.11±2.77	ND	17.20±2.76	26.68±3.19
PsO	29.84±0.84	29.79±1.25	ND	31.70±0.89	21.08±1.18	25.78±1.70	33.26±0	33.05±0.83	27.55±1.37	28.62±0.63	ND	28.09±0.92	ND	15.98±1.02	25.19±0.94
OA	31.48±0.54	31.35±0.13	ND	32.12±0	24.56±1.45	29.01±1.03	ND	ND	31.47±1.36	29.89±0	35.17±0	30.99±1.17	ND	15.03±1.08	28.28±1.33

	miR-378a-5p	miR-382-5p	miR-409-3p	miR-425-3p	miR-483-5p	miR-484	miR-499a-5p	miR-93-5p	miR-425-5p	miR-574-3p	miR-574-5p	miR-579-3p	miR-589-5p	miR-593-5p	miR-596
p-SpA	31.78±0.69	29.32±1.00	27.65±0.94	28.02±0.40	31.89±0.97	25.34±3.14	31.52±1.63	25.08±2.69	25.60±3.12	29.10±0.37	25.75±2.36	30.36±0.67	30.43±1.01	31.80±2.03	27.71±1.64

PsO	31.62±1.41	29.07±0.34	29.60±1.46	28.80±0.62	31.71±0.07	23.85±1.12	ND	24.10±0.75	23.91±0.84	29.82±1.32	24.30±0.35	ND	30.63±0.54	33.88±0.68	26.54±1.58
OA	ND	28.29±0.15	32.85±0	32.28±0	32.08±0	27.32±1.43	33.62±0	26.19±1.07	26.05±0.99	33.01±0	24.84±0.66	30.44±0	33.61±3.14	32.49±0	30.95±1.25
	miR-601	miR-216a-5p	miR-940	miR-34a-5p	miR-30b-5p	miR-375	miR-500a-5p	miR-144-3p	miR-1228-5p	let-7b-5p	let-7c-5p	let-7d-5p	let-7f-5p	let-7g-5p	miR-100-5p
p-SpA	ND	31.75±0	28.14±1.32	29.28±0.26	27.24±4.49	32.66±0.48	33.16±2.15	ND	29.16±0.68	22.24±3.19	28.85±3.49	25.12±3.77	ND	28.41±2.46	28.92±1.26
PsO	ND	ND	27.57±0.27	32.05±2.25	24.08±1.44	30.39±0.31	31.87±0	ND	28.99±0.44	20.53±0.74	29.21±1.17	23.04±1.24	ND	29.84±1.58	29.84±2.40
OA	ND	ND	ND	29.60±0.50	27.16±1.40	31.99±0	32.25±0	ND	30.07±1.05	22.26±0.93	32.58±2.20	27.63±1.92	ND	32.38±0.85	29.09±0
	miR-10a-5p	miR-10b-5p	miR-130a-3p	miR-130b-3p	miR-133a-3p	miR-133b-3p	miR-134-5p	miR-106a-5p	miR-141-3p	miR-146b-5p	miR-208a-3p	miR-17-5p	miR-18a-5p	miR-18b-5p	miR-192-5p
p-SpA	30.92±0.26	31.32±0	24.30±3.31	25.48±1.76	28.52±0.69	ND	28.26±0.46	27.58±1.10	27.89±1.08	27.57±2.06	ND	24.75±1.61	25.58±2.65	30.71±0.79	28.41±0.74
PsO	30.60±1.35	33.64±1.87	23.44±1.19	25.20±1.19	29.44±1.00	27.69±0	28.62±0.00	29.05±0.62	27.98±0.36	28.26±2.03	ND	25.14±0.37	25.05±1.24	30.16±0.46	29.18±0.56
OA	ND	ND	27.00±1.80	28.07±0	28.63±0	ND	28.31±0	29.20±1.15	ND	31.23±0.63	ND	27.34±0.25	28.54±0.98	31.38±0.08	28.93±0.58
	miR-195-5p	miR-196b-5p	miR-200a-3p	miR-200b-3p	miR-200c-3p	miR-20b-5p	miR-210-3p	miR-221-3p	miR-27a-3p	miR-29a-3p	miR-29c-3p	miR-30a-5p	miR-30d-5p	miR-30e-5p	miR-376c-3p
p-SpA	28.80±0.63	30.49±0	33.59±1.65	ND	31.05±0.59	30.90±1.29	27.40±0.45	25.96±2.96	24.56±4.00	26.43±3.66	23.68±1.58	28.95±2.24	27.27±4.28	27.51±4.34	29.62±0.93
PsO	30.78±0.86	ND	31.33±0	ND	ND	30.74±0.75	27.55±0.14	25.66±1.57	21.91±1.25	24.24±0.92	24.00±0.58	28.38±0.69	26.82±1.42	24.96±0.81	31.26±0.84
OA	30.03±0	ND	ND	ND	ND	33.11±2.10	27.71±0.93	29.89±1.63	24.98±1.51	26.44±0.64	24.75±0.88	31.40±0.40	28.94±0.54	26.80±0.99	ND
	miR-411-5p	miR-486-5p	miR-223-3p	miR-1-3p	miR-625-5p	miR-652-3p	miR-660-5p	miR-663a	miR-718	miR-7-5p	miR-760	miR-885-5p	miR-92a-3p	miR-95-3p	miR-193b-3p
p-SpA	26.87±0.95	21.61±2.60	22.61±4.40	29.53±1.23	27.99±1.98	26.93±4.39	31.27±1.44	29.16±1.78	32.85±0	30.29±0.51	31.77±1.35	32.23±1.04	22.50±3.33	ND	31.82±1.76
PsO	27.96±0.55	20.30±0.53	19.77±1.25	31.30±0.87	28.96±1.23	24.82±1.53	29.90±0.82	30.17±1.51	33.70±0	31.09±1.14	31.62±0	31.99±0.16	20.54±0.59	31.00±0	31.85±0.16
OA	ND	20.28±0.80	23.17±1.31	31.31±1.07	30.97±0	28.88±2.25	30.43±1.16	31.53±0.10	ND	32.20±1.19	ND	32.56±0.61	21.62±1.07	ND	29.47±0
	miR-29b-3p	miR-127-3p	miR-450a-5p	miR-518a-5p	miR-9-5p	miR-99b-5p	miR-128-3p	miR-31-3p	miR-320a	miR-199a-3p	miR-330-5p	miR-429	miR-125a-5p	miR-4306	
p-SpA	27.06±3.34	27.50±0.84	33.04±0.90	ND	33.05±0	24.69±0.54	26.95±1.88	27.10±0	25.73±4.11	26.36±4.22	30.07±1.92	29.08±0.70	25.71±2.43	32.50±2.29	

PsO	25.66±1.29	29.75±0.47	31.55±0.34	ND	31.49±0	25.67±0.98	26.91±0.87	ND	23.31±1.07	23.56±1.38	27.73±1.05	29.88±0.93	25.38±1.58	ND
OA	28.33±1.30	ND	ND	ND	ND	27.18±0	29.12±0.12	ND	24.64±1.26	28.50±1.74	31.06±0	31.86±0.60	28.17±0.78	ND

p-SpA, peripheral spondyloarthritis; PsO, psoriasis without clinical arthritis; OA, osteoarthritis; ND, not detectable.

Data are expressed as mean ± standard deviation
